# Dishonesty and research misconduct within the medical profession

**DOI:** 10.1186/s12910-020-0461-z

**Published:** 2020-03-18

**Authors:** Habib Rahman, Stephen Ankier

**Affiliations:** 1 Specialist Registrar in Cardiology and General Medicine, NHS, London, UK; 2 Independent Pharmaceutical Consultant, Middlesex, UK

**Keywords:** Scientific reproducibility, Medical ethics, History of medicine, Royal College of Physicians, Sociology of the medical profession

## Abstract

While there has been much discussion of how the scientific establishment’s culture can engender research misconduct and scientific irreproducibility, this has been discussed much less frequently with respect to the medical profession. Here the authors posit that a lack of self-criticism, an encouragement of novel scientific research generated by the recruitment policies of the UK Royal Training Colleges along with insufficient training in the sciences are core reasons as to why research misconduct and dishonesty prevail within the medical community. Furthermore, the UK General Medical Council’s own data demonstrates a historic inattentiveness to the ease with which doctors can engage in research misconduct. Suggestions are made as to how these issues can be investigated and alternative incentives for career advancement are adumbrated.

“The honour of belonging to the Royal Society is much sought after by medical men, as contributing to the success of their professional efforts, and two consequences result from it. In the first place the pages of the Transactions of the Royal Society occasionally contain medical papers of very moderate merit; and, in the second, the preponderance of the medical interest introduces into the Society some of the jealousies of that profession. On the other hand, medicine is intimately connected with many sciences, and its professors are usually too much occupied in their practice to exert themselves, except upon great occasions.”

*Charles Babbage, Reflections on the Decline of Science in England, and on Some of its Causes – Of the influence of the Colleges of Physicians and Surgeons (1830)*

## Introduction

This excerpt from Babbage’s famously cantankerous treatise continues to ring true almost 200 years later. Though a great deal of newspaper print, conference discussion and journal space has been dedicated to addressing the urgent concerns of scientific irreproducibility and outright fraud amongst full-time scientists [1–5] the reasons for research misconduct of medical doctors (hereon referred to simply as doctors) has attracted far less attention here in the UK.

After behaving dishonestly in the operation of clinical trials involving patients at his Lancashire GP practice, the erasure of Jerome Kerrane from the UK medical register in 2019 serves as another reminder, almost 10 years after Andrew Wakefield’s erasure from the medical register, of how the public image of doctors can be tarnished [6]. However, reflection on whether the perpetrators are cynical wildcards or our professional culture or institutional policies may be contributory remains to be seen. Conflicts of interest between researchers and drug and device companies have been discussed at length [7–10] but examination of the professional culture of medicine in the UK is uncommon, critical analysis of the training and regulatory institutions even less so. Richard Smith and Peter Wilmshurst are amongst the small number of critics of a professional culture that finds it difficult to accept the presence of dishonesty amongst its ranks and sometimes actively refuses to investigate it [11–13]. In 1993 Stephen Lock and Frank Wells published the first edition of the only book in print dedicated to medical research misconduct and have also made recommendations as to how the profession can be policed to prevent abuses of power [14]. The transfer of European Union clinical trials legislation into UK law in 2004 made significant deviations from the GMC’s *Good Medical Practice* illegal within the context of clinical trials. Notwithstanding this, there has been little to no advancement in regulating doctors’ forays into research.

## Historical context

The association between medicine and scientific theory is probably as old as medicine itself, given the need for a philosophy of health and disease in which to couch the pragmatic business of diagnosis and cure. For most of British history, the practice of healthcare was delivered by an admixture of practitioners trained via informal apprenticeships and university educated physicians. The Enlightenment for the medical profession was predominant, if not incipient in the early decades of the eighteenth century and the workings of a middle ground between empiricism and rationalism were soon underway. Though some scientific and technical advances were made in the eighteenth century, the proliferation of doctors seemed to make little difference to the onslaught of disease that afflicted the British people. Overall, doctors developed a public image that would be an outright embarrassment today.

“Satirists, cartoonists and commentators widely portrayed medicos as pompous asses, seeking to hide their ignorance behind a veil of hard names in dead tongues, rapaciously exploiting the helplessness of the sick … In short, everybody could see that medicine was hardly making much real progress towards the goal of rendering life safe and healthy, and there was a widespread perception that a malaise infected the medical profession itself. Indeed, medicine lacked any professional presence. The London colleges dwindled into insignificance and offered no leadership … [15]”

Unsurprisingly, university trained doctors looked for ways to further their name amongst the wealthy, redounding to the disrepute of the profession inchoate. The historical record demonstrates that a desire for wealth, prestige and celebrity was a motivation for many Enlightenment physicians to publish books and magazine articles, with features familiar to the medical profession of today:

‘The choice of the medical topic does not seem to have been important. Fresh graduates asked patrons, professors, and even friends for advice on subjects likely to forward their names in the world. And yet the content had to be creditable and avoid the pitfalls of precipitated self-promotion. The obvious difficulty was that would-be authors had little practical knowledge and no research infrastructure on which to fall back.’ [16]

## Modern day research misconduct

Circumstances for doctors have changed greatly since the nineteenth century, when competition with quacks, apothecaries and the fraternity made their income uncertain. Given that today’s doctors are comfortable both in their remuneration, social standing and job security, transgressions of public trust are indefensible. Though the Wakefield case engendered condemnation from the biomedical community worldwide, it shouldn’t be forgotten that his 1998 Lancet paper passed elite peer review standards, his career was unblemished until the late 1990s and similar breaches of trust are not rare in the medical community. One of the most egregious cases of research misconduct in the UK was exposed earlier in the 1990s when the distinguished gynaecologist Malcolm Pearce of St. George’s Hospital in London fabricated a report describing the transplantation of an ectopic pregnancy into a patient’s uterus to result in a live birth, as well as falsifying a clinical trial on 191 women with polycystic ovarian syndrome suffering from recurrent miscarriage [17]. Despite the high profile nature of Pearce’s case at the time and a formal statement from the respective journal declaring that both publications should be withdrawn, the clinical trial report’s abstract remained intact on the journal’s website for several years and the paper was subsequently cited in several papers as an example of fertility treatment [18–25].

Unfortunately the problem of faulty journal articles remaining within the research corpus is not rare and has been discussed, along with myriad other factors undermining the reliability of the scientific enterprise, in Richard Harris’ compelling book *Rigor Mortis*^14^. Since the early 2000s major concerns have been raised over the difficulty in reproducing many published scientific findings [26]. In 2015 a London symposium on the issue crystallised in a useful report published by the Academy of Medical Sciences [3], while the Royal Society’s more recent series of debates on scientific culture further emphasised the importance of scientific irreproducibility:

‘Narrow approaches to assessment based on publication metrics risk promoting an environment in which systemic pressures may incentivise individuals to compromise on the rigour and integrity of their research.’ [4]

Suggestions abound as to how to improve this culture to ensure that reliable, honest science is incentivised and for dishonest miscreants and incompetence to be discouraged [4, 27]. Criticisms have occasionally been scathing, such as Barbara Redman stating in 2013 - ‘there is a disconnect between ethical norms and the real world of science’, along with helpful suggestions for how the problems can be addressed [28]. It is through reflecting on the reproducibility crisis along with the pressure of publishing in top ranking journals that the unfortunate side effect of research misconduct within the scientific community has become better recognised [7].

## The reasons for research misconduct amongst doctors

Doctors involved in research misconduct are generally characterized as dishonest anomalies within a profession that is otherwise careful of its scruples, the so-called ‘bad-apple metaphor’ [28]. A corollary of this is a lack of the same reflective criticism that the scientific community is employing to examine dishonesty amongst its own ranks. The oft cited meta-analysis by Daniele Fanelli of the University of Edinburgh shows that around 2% of researchers (including doctors) admit to having falsified, fabricated or modified data for publication before, with 14% being aware of colleagues having done so [29]. Looking specifically at the UK, it is remarkable that only a single published study has specifically questioned doctors with respect to research misconduct. David Geggie, now a Consultant in Emergency Medicine, conducted a survey in 2000 asking hospital consultants whether they had ever performed or witnessed research misconduct. Including ‘softer’ forms of research misconduct, such as guest authorship, 5.7% of respondents admitted to having carried it out, while 4.1% of respondents expressed a willingness to selectively report data if it would enhance a grant application [30].

Importantly, 59.8% of respondents in Geggie’s study reported a sense of necessity in publishing papers in order to further their career. As Babbage identified, though post-Enlightenment medicine has an intimate connection with the sciences it is difficult for practicing doctors to find the time to get involved with medical research, let alone act as lead investigators on such projects. This is even truer today, with the cumbersome bureaucratic nature of existing medical practice and postgraduate medical training. Irrespective of these pressures, it is remarkable as to how rarely the assumption that doctors ought to be doing, rather than informing medical research has been questioned. In the words of the renowned medical statistician Doug Altman:

‘The length of a list of publications is a dubious indicator of ability to do good research; its relevance to the ability to be a good doctor is even more obscure.’ [31]

This then begs the question as to why this tendency exists. Given the lack of any clear relationship between conducting a research project and the day-to-day grind of diagnosing disease and treating patients, why is there such a pressure on doctors to author original research publications?

Fortunately a major contributor to this incentive is easier to identify and change than much of the cultural afflictions of the scientific establishment. As evidenced by the person specification documents on the NHS specialty training website, the Royal Medical Colleges (with the exception of the Royal College of General Practitioners) encourage applicants to conduct scientific research [32]. Most of the Royal Colleges do not make their exact application scoring criteria public but the Royal College of Physicians is exceptional in this regard. Top marks are awarded for those candidates who can claim: ‘I am first author, or joint-first author, of two or more PubMed-cited original research publications (or in press)’, with no importance placed on whether or not the subject of this research is relevant to the job in question [33]. Given the emphasis placed on the ‘publish or perish’ culture that encourages scientific misconduct and irreproducible research amongst scientists, it stands to reason that the incentive scheme employed by the Royal College of Physicians would have a similar effect.

It is, of course, not necessarily due to wilful misconduct that a doctor’s research be found to be irreproducible. The ‘hoaxing, forging, trimming and cooking’ that Babbage referred to comprises, one hopes, of only a minority of low quality publications, but the lack of access to adequate preparatory training that scientists have reported is of even greater relevance to the doctor who wishes to foray into research [3, 27]. The reflections of the scientific community in this regard make a salutary, though no doubt unintended, inference to a further contradiction in the doctor’s desire to front research projects. If full-time scientists feel underprepared to conduct research, it seems counterintuitive for a doctor to lead the charge [34]. Indeed the Royal College of Physicians’ 2016 study on doctors’ involvement with research showed that, when averaged across all grades of experience, insufficient knowledge in statistical skills was the most common impediment to performing research. Other important barriers included not knowing who could act as a research mentor and, particularly at more junior levels, insufficient expertise in the area in which research might be considered [35]. This echoes the findings of the independent inquiry commissioned by The Royal College of Obstetricians and Gynaecologists following the Malcolm Pearce case, as he was able to convince a doctor he was supervising for a PhD to unwittingly help him with his fraud:

‘The evidence presented to us highlighted the need for appropriate research training in obstetrics and gynaecology … Such training should include a basic understanding of research ethics, study design and analysis and an understanding of the processes involved in publication and the responsibilities of authors. Trainees should also be aware of what constitutes appropriate training and supervision in research.’ [36]

Regrettably, almost 25 years later, mandatory preparatory training is yet to be established in the UK. Another important finding of the Royal College of Physicians’ 2016 study was that doctors frequently considered performing research as a means to boost their résumé. However, the College’s interpretation of this appears to be in the positive.

“ … for [junior doctors] extrinsic motivators were reported to be much more important. The most clear example of this is for the statement ‘it enhances my CV or publications record’ … This seems to indicate that being involved in research is seen as a good boost to the employability for those still in training.” [22]

This is unsurprising given the incentive structure in place, and as described later, the North American experience demonstrates that this can, perhaps predictably, encourage bad behaviour.

## The legal and professional frameworks surrounding research misconduct

Since they are bound by GMC codes of practice, are doctors held to a different professional standard to scientists? In essence, yes. The GMC documents *Good medical practice* and *Good practice in research* clearly describe honesty as ethically binding to all aspects of medical and research practice, so evidence of breach of these guidelines could be punished with GMC sanctions [37, 38]. However although investigators involved in clinical trials, such as Jerome Kerrane, are legally bound by the UK Clinical Trials Regulations, there is no legislative framework criminalising research misconduct outside of a clinical trial. Hence a doctor who fabricates a research article or case report could be sanctioned by the GMC but would not have broken any UK law. Even in this regard, GMC sanctions where research misconduct was a salient factor are very rare, with only 15 cases in the past 13 years and a lacklustre record on issues of research misconduct in general [39, 40].

## The relationship between research misconduct and medical specialisation

While attempts to study dishonesty and misconduct amongst UK doctors total just 1 survey published in 2000, 3 more recent surveys of US specialty training applications have elicited sobering discussions amongst the American medical community. The authors checked the published journal articles and abstracts stated on candidate’s urology, ophthalmology and neurosurgery training programme applications. The results were startling, revealing misrepresentation and sometimes complete fabrication of articles, with rates ranging from 5 to 45% [41–43]. It is of no surprise that commentary on these findings mentioned the competitiveness of these specialties as partial explanations for the transgressions, with similar findings from a Canadian study published in 2015 [44]. It is long overdue for UK specialty training applications to be analysed in a similar manner. Furthermore embarking on an MD or PhD programme, often funded with public or charitable funds, has become extremely common for highly competitive specialties such as surgery or cardiology. Given the lack of preparatory training and questionable incentives of many doctors carrying out such research, the reproducibility of their results compared with work done by full-time scientists would be especially worthy of examination.

## Preventing research misconduct

Calls for research misconduct to be legally prohibited have been made for years [45] and such legislation should perhaps be designed with special attention to doctors, given the extraordinary access they have to patients’ bodies and intimate information. An independent regulatory body dealing with all issues of scientific integrity could help to protect researchers who raise good faith confidential concerns from retaliatory lawsuits launched by powerful pharmaceutical and biotechnology companies [46]. However the medical profession first needs to acknowledge its substantial contribution to the volume of biomedical research publications and the possibility of dishonesty or incompetence in this research having a significant effect. Despite numerous scientific conferences and reports dedicated to the issue of misconduct, no such self-examination has been demonstrated by the Royal Medical Colleges or the GMC. It is noteworthy that the most recent World Conference on Research Integrity had no practising doctors as speakers, while a research study from a Malaysian group suggests that academics in healthcare departments were more likely to be involved with research misconduct than other academics [47]. Liang, Mackey and Lovett at the UCSD School of Medicine incisively argue that the negative effects of research fraud are incalculable due to the reliance of low-income countries on our research to guide the development of their own efforts. Describing the damaging effects of a dishonest culture on the mind of the developing clinician:

‘ … students and trainees do not push back against this system because of the reasonable fear of recrimination within the culture, and then over time and long hours simply become acculturated to it as a matter of survival. Yet a culture where senior academic physicians are unchallenged, unethical, and reward dishonest medical trainee applications supports a never-ending cycle of inculcation of corrupting influences in each generation of academic medicine.’ [48]

Discussions of medical education’s role in preventing research misconduct are frequently misplaced since the reasons for refraining from research misconduct barely extend beyond any normative conception of ethical behaviour and distracts from the doctor’s agency to behave ethically [49]. Fenton and Jones in their otherwise helpful 2002 paper state that:

‘The preponderance of misconduct occurs because many authors are not informed on ethics, as these issues have generally not been addressed in medical undergraduate or postgraduate education.’ [50]

The truth of this statement is undermined by the absence of any advanced ethical framework required to understand that dishonest research activity is unethical, let alone its incommensurability with the role of a doctor. In the words of the influential British medical ethicist Thomas Percival:

‘Let both the Physician and Surgeon never forget that their professions are public trusts, properly rendered lucrative whilst they fulfil them, but which they are bound by honour and probity to relinquish as soon as they find themselves unequal to their adequate and faithful execution.’ [51]

Whether medical education academes are best placed to instruct on these virtues remains unclear, particularly in light of the striking research findings of Anthony Artino Jr. and colleagues, revealing that over 90% of the healthcare education researchers in their international survey admitted to having partaken in some form of questionable research practice, including 49.5% of respondents confessing to have cited papers that they had not read [52]. Though fora to allow researchers to discuss research ethics are likely to be helpful, it is unknown as to who would be best placed to model the attributes espoused by Percival, or whether the virtues can be inculcated via professional or academic programs at all [53].

## Conclusion

The code of silence that protects senior doctors from scrutiny is rarely discussed in the UK and the GMC could help break this by issuing more sanctions on doctors who have acted in a wilfully dishonest fashion [54]. There is a compelling need for the Royal Medical Colleges to assess the degree of misrepresentation in doctors’ job applications and should reformulate how medical trainees are incentivised. Demonstrating involvement with patient safety improvement or already existing research projects would be a much more constructive incentive than that which not only allows research misconduct, but appears to encourage it [31]. Though the medical profession still secures the trust of the majority of the British people, there is some suggestion that this has been declining [55]. A meaningful discourse on how our training and reward systems should be improved would help prevent future generations of doctors from pursuing dishonest opportunities to advance their careers and would have a positive effect on public trust in the medical profession, a relationship that we still rely on to serve our patients effectively.

## Data Availability

All data referred to in the manuscript is publicly accessible.
